# Salaries at the General Hospital, Wellington

**Published:** 1920-08-21

**Authors:** 


### Nursing Conditions in New Zealand.

### Salaries at the General Hospital, Wellington.

THE public hospitals in New Zealand are realising that if they wish to make the nursing service more attractive it is time to make it more remunerative. Recently the Wellington Hospital Committee reported to the Board "that up to the present the Matron has been fortunate in always being able to fill vacancies on the nursing staff, but the waiting-list is lessening yearly, and as a consequence the selection of suitable candidates is increasingly difficult. It is understood that there is a similar diminution of applicants in other centres, and it is considered general throughout the hospital world. If this shortage were made known application might now be made by the class of woman who would be an acquisition to the nursing profession. The ideal of nursing should appeal to those who have the vocation for this work."

The average number of occupied beds per diem last year was 380, out-patients numbered 7,836, with 78,504 visits. The nursing staff numbered 145 (33 certificated and 112 probationers). The new scale of salaries adopted, which came into force from April 1 last, is as follows: Probationers, £30 first year; £40 second year; £50 third year. Nurses, £90 first year after training; £100 thereafter. Sisters, £100 first year; £120 second year; £130 thereafter. (An extra allowance of 10s. 6d. per week is payable to the night sister.) Home sister, £150, rising to £170, with two annual increments of £10 each. Assistant matron, £175 first year; £185 second year; £195 thereafter. Matron, £300, rising to £350 by two instalments of £25 per annum. In considering these salaries it should be remembered that board and residence, uniform, laundry, and medical attention are estimated to be worth £88 per annum. The Wellington Hospital Board is to be commended for establishing a lead to the other Boards in New Zealand.

The Nurses' Registration Act has been in force in New Zealand for many years, and nurses who have obtained their hospital certificates have no difficulty in passing the State examination. The eight hours a day system for nurses prevails in public hospitals in New Zealand, and it is specially enacted that in any hospital of over 100 beds the hours of any uncertificated nurses shall not exceed fifty-six in any one week.

In the Auckland, Wellington, and Dunedin hospitals sisters have one day monthly, one Sunday in six, and three days at the end of six months respectively, in addition to a half-day weekly at the two first-named hospitals. Nurses have three weeks annual leave. Christchurch gives annual leave only, and it needs to bestir itself in the matter of the monthly and weekly off-duty time or it may find itself confronted with a shortage of nurses.

The sisters and nurses who have been absent with the New Zealand Expeditionary Force have now returned, and those who have not been retained in the military hospitals are either being absorbed in the public hospitals, private hospitals, nursing homes, or have taken up private nursing.

